# BTK Inhibitors in Chronic Lymphocytic Leukemia: Biological Activity and Immune Effects

**DOI:** 10.3389/fimmu.2021.686768

**Published:** 2021-07-01

**Authors:** Marzia Palma, Tom A. Mulder, Anders Österborg

**Affiliations:** ^1^ Department of Oncology-Pathology, Karolinska Institutet, Stockholm, Sweden; ^2^ Department of Hematology, Karolinska University Hospital, Stockholm, Sweden

**Keywords:** BTK inhibitors, ibrutinib, leukemia, off-target effects, immune cells, infections, vaccination

## Abstract

Bruton´s tyrosine kinase (BTK) inhibitor (BTKi)s block the B-cell receptor (BCR) signaling cascade by binding to the BTK enzyme preventing the proliferation and survival of malignant and normal B cells. During the past decade, the clinical use of BTKis for the treatment of B-cell malignancies has exponentially grown, changing the treatment landscape for chronic lymphocytic leukemia (CLL) in particular. At present, three different covalent BTKis, ibrutinib, acalabrutinib and zanubrutinib, are FDA-approved and many new inhibitors are under development. Despite having remarkable selectivity for BTK, the first-in-class BTKi ibrutinib can also bind, with various affinities, to other kinases. The combined inhibition of BTK (“on-target” effect) and other kinases (“off-target” effect) can have additive or synergistic anti-tumor effects but also induce undesired side effects which might be treatment-limiting. Such “off-target” effects are expected to be more limited for second-generation BTKis. Moreover, the blockade of BCR signaling also indirectly affects the tumor microenvironment in CLL. Treatment with BTKis potentially impacts on both innate and adaptive immunity. Whether this affects infection susceptibility and vaccination efficacy requires further investigation. Here, we summarize the available knowledge on the impact of BTKis on the immune system and discuss the possible clinical implications. Indeed, a deeper knowledge on this topic could guide clinicians in the management and prevention of infections in patients with CLL treated with BTKis.

## Introduction

Chronic lymphocytic leukemia (CLL) is characterized by the accumulation of mature monoclonal CD5^+^ B lymphocytes in the secondary lymphoid organs, bone marrow and peripheral blood. Signaling through the B-cell receptor (BCR) is central in CLL and Bruton’s tyrosine kinase (BTK) is a vital component of the BCR signaling pathway (1). BTK inhibitor (BTKi)s block the BCR signaling cascade by binding to the BTK enzyme, hence preventing the proliferation and survival of malignant and normal B cells. Indeed, BTK is essential for the activation of pathways which promote lymphocyte survival, such as the nuclear factor kappaB (NF-κb) pathway (2), and regulates chemokine secretion and B-cell adhesion by activation of phospholipase Cγ2 (PLCγ2) (3).

The first-in-class BTKi ibrutinib, which inactivates BTK by binding covalently to cysteine 481 in the ATP-binding site of the enzyme in an irreversible manner, is FDA-approved for the treatment of CLL, mantle cell lymphoma (MCL), Waldenström’s macroglobulinemia, marginal zone lymphoma, and graft-versus-host disease. At present, two additional covalent BTKis, acalabrutinib and zanubrutinib, are FDA-approved for the treatment of MCL and CLL. Many new inhibitors, which bind reversibly, non-covalently, to the BTK are under development. The structure-function relationships of covalent and non-covalent BTKis are discussed by Zain et al. in the same issue of this journal.

In the past years, BTKis have been increasingly used for the treatment of B-cell malignancies, significantly changing the treatment algorithm for CLL in particular.

Despite having remarkable selectivity for BTK, ibrutinib can bind to other kinases with various affinities. Among these, interleukin-2-inducible T-cell kinase (ITK), which is highly expressed in T cells, and three epidermal growth factor receptor (EGFR) family kinases: EGFR, ErbB2/HER2 and ErbB4/HER4. The combined inhibition of BTK (“on-target” effect) and other kinases (“off-target” effect) can have additive or synergistic anti-tumor effects but also induce undesired side effects, such as atrial fibrillation (AF) and bleeding.

The two second-generation BTKis, acalabrutinib and zanubrutinib, were designed to more selectively bind to cysteine 481 in the kinase domain and therefore have less “off-target” effects than ibrutinib. Phase 3 clinical trials are ongoing to compare the efficacy and toxicity profile of acalabrutinib and zanubrutinib with ibrutinib in CLL.

Besides “on-target” and “off-target” activity, there is, at least in CLL, convincing evidence that the anti-tumor effect of ibrutinib is related to the indirect effect that the blockade of the BCR-signaling has on the tumor microenvironment (TME) (4). In CLL, the TME is supporting the tumor cell survival and growth by various mechanisms including triggering of BCR signaling and immune anergy, which leads to immune deficiency and susceptibility to infections. Treatment with BTKis potentially impacts on both innate and adaptive immunity, but to what extent this affects infection susceptibility in patients with CLL is still under investigation.

Here, we summarize the available knowledge on the biological activity of BTKis on immune cells, trying to dissect which of the effects can be attributed to BTK-inhibition itself (“on-target” activity), which indirectly result from BTK-inhibition suppressing the cross-talk within the TME (“on/off-target”), and which are truly due to the inhibition of kinases other than BTK (“off-target”).

## Anti-Tumor Effects of BTK Inhibitors in CLL

The active occupancy of the ATP-binding site of BTK by BTKis inhibits the subsequent phosphorylation of BTK, PLCγ2, AKT and ERK abolishing the BCR signaling downstream of BTK both *in vitro *and *in vivo*. In CLL, the BCR signaling pathway is constitutively activated, in immunoglobulin heavy-chain variable (IGHV) mutated cases in particular (5), and the anti-tumor effect of BTKis firstly depends on the direct inhibition of BTK (6). Indeed, after inhibition of the BTK, pathways involved in CLL cells survival and expansion, such as the NF-κb pathway (7) as well as BAFF-R signaling (8), cannot be activated. Chemokine secretion, specifically CCL3 and CCL4, and adhesion of B cells, is also inhibited (9). The degree of BTK inhibition is traditionally measured by assessment of target occupancy, which has been evaluated in phase 1 studies. Available data suggest that sustained target occupancy during acalabrutinib treatment is necessary for the down-regulation of pathogenic pathways and to slow down BTK re-synthesis in CLL patients (10). However, whether the degree of BTK occupancy impacts on the clinical outcome is still unknown.

Ibrutinib, the pioneer of BTKis, exhibits remarkable selectivity for BTK. However, its anti-tumor effect likely also depends on the indirect effect that the blockade of the BCR-signaling has on the TME (“on/off-target”). Indeed, the TME in CLL plays a major role in sustaining tumor cell survival and growth in the bone marrow and/or lymphoid tissues (11). This is further supported by the limited direct pro-apoptotic activity observed when purified CLL cells are incubated *in vitro* with ibrutinib (12), while apoptosis can be induced through mobilization of the leukemic cells from the lymph nodes *in vivo* (7, 13). Upregulation of the survival protein Bcl-2, can be induced, as an example, by T cells through soluble factors such as IL-4 and IFN-γ (14, 15), or directly through CD40-CD40L interaction (16), which is a key proliferative signal for CLL cells. Recently, it was shown that miRNAs can actively participate in the crosstalk between CLL cells and T cell signals and facilitate the co-occurrence of BCR and CD40 signaling activation (17).

Chemokine receptors and adhesion molecules expressed on CLL cells are involved in the CLL-cell homing to the lymphoid organs. As an example, the CXCR4 chemokine receptor is highly expressed on the surface of CLL cells in the peripheral blood and mediates CLL cells chemotaxis and migration in response to CXCR4 ligand stromal cell-derived factor 1 (SDF-1α, CXCL12) produced by NLC. BCR engagement on CLL cells induces increased expression of these molecules (9) and it is therefore not surprising that BTK-inhibition by ibrutinib affects tumor cells-TME cells interactions, e.g. impairing CXCR4 signaling and therefore inhibiting cellular adhesion (18, 19). Indeed, shortly after start of ibrutinib treatment, a temporary increase in the absolute lymphocyte count, so-called “redistribution lymphocytosis”, is observed in patients with CLL (13, 20), reflecting the release of tumor cells from the lymphoid organs to the peripheral blood (4). The down-regulation of the CXCR4/CXCL12 axis also leads to the down-regulation of both CD20 and of the anti-apoptotic protein Mcl1 *in vivo* (21).

Finally, nine other kinases which have a corresponding cysteine residue in the ATP-binding site as BTK, are also likely affected by ibrutinib. These include four TFK members (ITK, TEC, BMX and RLK/TXK), three EGFR family kinases (EGFR, ErbB2/HER2 and ErbB4/HER4) and two other kinases, BLK and JAK3. The inhibition of these other kinases (“off-target” effect) can contribute to ibrutinib´s anti-tumor activity but also induce side effects which might be treatment-limiting.

Historically, the treatment of choice for CLL has been chemoimmunotherapy but the prognosis for high-risk patients, i.e. with relapsed/refractory (R/R) or del17p/*TP53*-mutated disease has been poor (22). However, during the last decade targeted therapies including BTKis, B-cell lymphoma 2 (BCL2) antagonists and phosphoinositide 3-kinase (PI3K) inhibitors entered the therapeutic scene and significantly improved the overall outcome for patients with *TP53* aberration and advanced-phase CLL (23–25).

Ibrutinib is now a cornerstone in high-risk patients (23) and often recommended as first-line treatment particularly in IGHV unmutated cases in the US (26–28). Overall response rates generally exceed 80% in trials and outside in both treatment-naïve and R/R patients but complete response rates are less than 10% (23, 27, 29, 30). Treatment duration is indefinite, i.e. the patients stay on treatment until disease progression or unacceptable toxicity occurs. This latter, most commonly including AF, infections, pneumonitis, bleeding and diarrhea, led to treatment discontinuation in approximately 10% of the patients in clinical trials (31).

## Immunomodulatory Effects of BTK Inhibitors

Beyond its role in the development and functioning of B cells, BTK seems also to play a key role in innate immunity, being expressed in myeloid and other innate immune cells and regulating a number of immunological signaling networks within cells of the innate immune system (32). Moreover, as mentioned above, the selectivity for BTK of ibrutinib in particular is far from being absolute, which can result in “off-target” effects on immune cells of both the innate and the adaptive immune system. Therefore, the immunomodulatory effects of BTKis can depend both on “on-target” on immune cells other than B cells and “off-target” effects.

### Effect on Innate Immunity

Ibrutinib seems to hamper the cytotoxic activity of NK cells (33), while zanubrutinib does not seem to impair NK cell function to the same extent. This is most likely due to the inhibition of ITK by ibrutinib, which is much weaker with zanubrutinib, as demonstrated in patients with MCL (34). ITK also mediates downstream signaling of the Fc receptor (FcR) (35), which might explain why ibrutinib reduced antibody-dependent cellular cytotoxicity (ADCC) by NK cells (36).

BTK is important for the development of neutrophils (37) and mediates signaling through Toll-like receptors and the FcR as well as activation of the NLRP3-inflammasome in macrophages (32, 38–40) and DC (41, 42).

As the most potent antigen-presenting cells (APC), DC play a crucial role in bridging innate and adaptive immunity, thereby facilitating immunological memory, which is the cornerstone of vaccination. Hepatocyte growth factor (HGF) and T cell Ig and mucin protein-3 (TIM-3)-mediated BTK activity can suppress the NF-κB pathway in DC, which inhibits their activation and maturation (43, 44). On the other hand, ibrutinib-treated DC have been shown to promote T-cell proliferation and drive a Th17 response (45).

It has also been demonstrated that both ibrutinib and acalabrutinib reduce the phagocytosis and secretion of inflammatory cytokines by monocytes and macrophages in response to fungal stimuli (46, 47), which could contribute to the susceptibility to invasive fungal infections (aspergillosis among others) observed in patients treated with ibrutinib (48–50). Furthermore, ibrutinib seems to reduce the expression of chemotactic factors that tumor-infiltrating macrophages (TAM), or nurse-like cells (NLC) in CLL, use to attract and protect CLL cells (51). Myeloid-derived suppressor cells (MDSC), another type of tumor-promoting myeloid cells, express BTK and it has been shown that ibrutinib blocks their development and immunosuppressive function (52). It has been observed that the expression of PD-L1 on MDSC decreases during zanubrutinib treatment (53).

Mast cells express BTK and other TEC family kinases (54). BTK, ITK and TEC are important for the degranulation and cytokine production of mast cells upon FcR stimulation in mice (55, 56) and ibrutinib has also been shown to reduce these functions in human cells (38). Although basophils and eosinophils are not so abundant, they play a significant role in the immune response against infections. The effect that BTKi might have on these cells deserves therefore to be studied more thoroughly.

Natural Killer T (NKT) cells and γδ T cells combine features that are characteristic for innate and adaptive immunity. Innate lymphoid cells (ILC) are primarily tissue resident innate immune cells that descend from common lymphoid progenitors. Ibrutinib seems to hamper degranulation of γδ T cells (57). Even if relatively scarce, these cell types are important in the early response against pathogens. Whether they are influenced by BTKis is a largely uncharted territory of research.

### Effects on Adaptive Immunity

Hypogammaglobulinemia is frequently observed in patients with CLL, with varying prevalence (20–70%) in different reports, and is more evident in progressive disease. Its severity correlates with infection risk (58), thus justifying immunoglobulin (Ig) replacement therapy. The etiology of hypogammaglobulinemia is not elucidated, but likely due to a perturbation of the T cell/B cell interaction induced by the cancer cells (59).

In the study by Sun et al., normal B cells seemed to increase over time during ibrutinib treatment, more significantly in treatment-naive patients compared to R/R ones. However, B-cell numbers remained abnormally low compared to healthy individuals. Despite the fact that BTK-deficiency in patients with X-linked agammaglobulinemia (XLA) leads to loss of circulating mature B cells and serum Igs, no significant decrease in IgG levels has been reported in patients during the first 6 months of ibrutinib treatment, while it became evident after 12 months (60). However, an increase of IgA levels over time has been reported by different studies (20, 29, 60), so at least a partial reconstitution of humoral immunity seems to occur.

Abnormal T-cell subset distribution and function is known in CLL, partly correlating with tumor burden and previous anti-cancer treatment (61). Skewing of the T-cell repertoire seems to occur early in the course of the disease, which has suggested that a positive selection of tumor-specific T-cell clones occurs (62).

The effects of ibrutinib on the T-cell compartment have been reviewed by Mhibik et al. (63). Even if the findings regarding the changes in T-cell numbers, including major subsets, during ibrutinib treatment have been controversial, with both reports on decrease (64, 65) and increase (66), the available evidence seems to point out that T cells decrease in parallel with the reduction of the tumor burden (64, 65, 67). Indeed, T cells are usually abnormally elevated in R/R CLL patients (61), chronically activated by tumor antigens. This might explain why T cells initially remain stable or increase during the first weeks of treatment when persistent lymphocytosis is observed, and then decrease later. However, no decrease in CD4^+^ and CD8^+^ T-cell numbers was seen after treatment with either acalabrutinib (66, 68) or zanubrutinib (53).

Proliferating T cells and expression of activation markers and immune checkpoints (PD-1, CTLA-4) also decreased both after treatment with ibrutinib (64, 65, 69), acalabrutinib (68) and zanubrutinib (53), also likely reflecting the decrease in tumor burden.

With regard to the T-cell receptor (TCR) diversity, discordant findings have been reported. In one study, an increase in TCR diversity was reported after 12 months of treatment ibrutinib with or without rituximab in patients with R/R CLL, positively correlating with clinical response and lower infection rates (67), while in another a more clonal repertoire was noted in patients responding to ibrutinib (70).

In T cells, ITK is highly expressed and blocked by ibrutinib in a BTK-independent manner (71). ITK plays a central role in T-cell maturation and differentiation into type 2 T-helper (Th2) effector cells. It has been shown that differentiation and migration of T cells to the lungs is compromised in ITK-knockout mice (72). By blocking ITK, ibrutinib has the potential to selectively decrease Th2 cell numbers causing Th1 skewing (69). Indeed, several studies have reported the reduction of some Th2-cytokines in ibrutinib-treated patients (64, 66, 67, 73). However, no change in Th1/Th2 polarization was observed in patients treated with acalabrutinib (66) or zanubrutinib (53), supporting the idea that ITK inhibition is central in Th skewing. Moreover, a decrease in Th17 cells and related cytokines was also observed (64, 67), as well as in the numbers of regulatory T cells (Tregs) in the first weeks of treatment (73), which is also supposed to be mediated by ITK inhibition. An increase in Th17 cells number was also observed in R/R patients treated with both acalabrutinib (66) and zanubrutinib (53). The immunomodulatory effects of BTKis are summarized in **Table 1**.

**Table 1:** Immunomodulatory effects of BTK inhibitors.

Cell type	Biological function	Impact of BTKi on cell number/function (mediated by)			
		ibrutinib	acalabrutinib	zanubrutinib
				
**ADAPTIVE IMMUNE SYSTEM**				
T cells					
CD8^+^	anti-tumor cytotoxic activity	decrease	no change	no change	
CD4^+^	helper function in Ag-presentation	decrease	no change	no change	
Th1	anti-tumor immune response	increase (RLK)?	no change	no change	
Th2	impairment of anti-tumor immune response	decrease (ITK)	no change	no change	
Th17	immunosuppression/immune evasion	decrease (ITK)	no change	decrease (?)	
Tregs	immunosuppression	decrease (ITK)	N/A	N/A	
B cells	IgA production	increase	N/A	N/A	
	IgG production	decrease	N/A	N/A	
**INNATE IMMUNE SYSTEM**				
NK	Cytotoxic activity	decrease (ITK)	N/A	N/A	
	ADCC	decrease (ITK-mediated signaling of the FcR)	N/A	N/A	
Phagocytes	engulf and digest pathogens	N/A	N/A	N/A	
Neutrophils	degranulate antimicrobial factors	decrease (BTK; TEC)?	N/A	N/A	
Monocytes/Macrophages/DC	Ag presentation	decrease (BTK *via* TLR, FcR and NLRP3-inflammasome). TEC?	N/A	N/A	
	phagocytosis and secretion of inflammatory cytokines in response to fungal stimuli	decrease (BTK)	decrease	N/A	
TAM (nurse-like cells)	expression of chemotactic factors for CLL cells	decrease	N/A	N/A	
MDSC	Tumor-promotion, immunosuppression	decrease (BTK)	N/A	decrease (BTK)	
Mast cells	degranulation and cytokine production in response allergens and parasites	decrease (BTK, ITK, TEC)	N/A	N/A	
basophils		N/A	N/A	N/A	
eosinophils		N/A	N/A	N/A	
NKT	early response	N/A	N/A	N/A	
γδ T cells	degranulation	decrease	N/A	N/A	

Ag, antigen; Th1, type 1 T-helper; Th2, type 2 T-helper; Tregs, regulatory T cells; Ig, immunoglobulin; NK, natural killer; ADCC, antibody-dependent cellular cytotoxicity; FcR, Fc receptor; DC, dendritic cells; Ag, antigen; TLR, Toll-like receptors; TAM, tumor-associated macrophages; MDSC, myeloid-derived suppressor cells; NKT, Natural Killer T; N/A, not assessed.

## Impact of BTK Inhibitors on Infection Susceptibility

In patients with CLL, recurrent infections are a major cause of morbidity and mortality. The high infection rate is due a number of immune defects inherent to the disease, consisting of hypogammaglobulinemia, abnormal T-cell function and neutropenia due to bone marrow infiltration, and further aggravated by cytopenias induced by CLL treatment (59). Hence, the risk of infectious complications increases with disease progression and the spectrum of the pathogens also expands, including, in addition to encapsulated bacteria mostly seen in patients with early, untreated CLL, more opportunistic agents such as *Pneumocystis jirovecii* and mycobacteria in the advanced disease setting (74).

Despite the high overall response rate and disease control achieved with ibrutinib, infections are not absent during long-term treatment with ibrutinib. By a systematic review of prospective trials with ibrutinib for the treatment of lymphoid malignancies, it was found that 56% patients treated with single-agent ibrutinib experienced an infectious complication of any grade. Grade 3-4 infectious adverse events occurred in 26% of the patients; of these, 13% were pneumonia (75). Of notice, the incidence of fungal opportunistic infections was in the range of 3 to 6% in reported series of patients with lymphoid malignancies, mainly CLL (76–78).

In patients with CLL during treatment with ibrutinib, most commonly respiratory tract infections, gastrointestinal/genitourinary and skin infections are observed (60). Higher infection rates have been reported in R/R patients, with infections ≥ grade 3 reported in more than 30% of the patients (20, 23) compared to 10-20% in those previously untreated (29, 30, 79). However, the incidence of infections seems to be highest during the first months of treatment to decrease over time (20, 60, 79).

In the study by Sun et al., the infection rate was significantly higher during the first 6 months of ibrutinib treatment, especially in previously treated patients compared to those receiving ibrutinib as first-line. As discussed above, in this study, despite normal B cells apparently increasing over time, IgG levels decreased, but IgA increased and a significant correlation was observed between increase in IgA levels at 12 months of treatment and infection rate, independent of previous treatment history (60).

Whether increased infection susceptibility is a class-effect or there are any differences between the different BTKis remains to be elucidated. Since it likely depends on the effect on different pathways and not only on BTK inhibition, it could be speculated that treatment with more selective BTKis, such as acalabrutinib, not affecting the ITK, could result in lower infection rates. However, in the largest phase 3 acalabrutinib trial in the R/R setting, grade ≥3 infectious complications occurred in 23% of the patients (68). **Table 2** summarizes in a simplified way how the effect of BTKi treatment on the different immune cell types can potentially affect susceptibility to different kinds of pathogens.

**Table 2:** Potential impact of treatment with BTK inhibitors on infectious susceptibility.

Cell type	Biological function	BTKi impact on cell number/function	On-target effect	Off-target effect	Potentially increased risk of infection by
**ADAPTIVE IMMUNE SYSTEM**					
T cells					
CD8^+^	cytotoxic activity	decrease		X	virus, bacteria
CD4^+^	helper function in Ag presentation	decrease		X	virus, bacteria
Th1	promotion of cellular immunity	increase/decrease		X	intracellular pathogens
Th2	promotion of humoral immunity	decrease		X	Extracellular pathogens, parasites
Th17	promotion of tissue inflammation, neutrophils recrutiment	decrease		X	extracellular bacteria and fungi
B cells	IgG production		X		bacteria, opportunistic pathogens
**INNATE IMMUNE SYSTEM**					
NK	Cytotoxic activity	decrease		X	Viruses, intracellular bacteria
	ADCC			X	
Neutrophils	Ag recognition	decrease	X		Bacteria, fungi
	maturation		X		
	degranulation		X		
Monocytes/Macrophages	Ag presentation	decrease	X	X (?)	Bacteria, fungi
	phagocytosis and cytokine secretion		X		
Mast cells	degranulation and cytokine production	decrease	X	X	parasites
γδ T cells	degranulation	decrease	?	?	

Ag, antigen; Th1, type 1 T-helper; Th2, type 2 T-helper; Ig, immunoglobulin; NK, natural killer; ADCC, antibody-dependent cellular cytotoxicity; Ag, antigen.

## Effect of BTKI on Vaccination Efficacy

Suboptimal response to vaccination has been reported in patients with CLL, even in those with indolent and previously untreated disease (80). In general, seroconversion in CLL patients negatively correlates with previous treatment. Following treatment with anti-CD20 monoclonal antibodies, the serological response to H1N1-influenza vaccination was absent for at least 6 months following exposure, recovering 12 months after (81).

Even if not all Ig subclasses seem to decrease in CLL patients during treatment with ibrutinib (20, 60), probably reflecting the persistence of long-lived plasma cells generated in response to previous infections and vaccinations, it is assumable that long-term BTKi treatment might impair the ability to mount an effective immune response following vaccination, since B-cell maturation relies on functional BTK.

In two studies evaluating the seroconversion rate after seasonal influenza vaccine in patients treated with ibrutinib, this was found to be as low as 7% after standard dose (82) and 26% after higher dose (83).

Pleyer et al. recently reported on the outcome of vaccination with the adjuvanted recombinant hepatitis B (HepB-CpG) and zoster (RZV) vaccines in CLL patients treated with BTKi compared to untreated patients. While no difference was observed between the two patient groups with regard to the serological response to RZV vaccine (41% *vs* 59%), the *de novo* humoral response to the HepB-CpG vaccine was significantly lower in BTKi-treated patients (3.8% *vs* 28%) (84).

Another study (85) recently confirmed the immunological efficacy of vaccination with adjuvanted recombinant varicella zoster glycoprotein in CLL patients during front-line therapy for ≥3 months with BTKi. Humoral and cellular responses were observed in 75% and 78% of the patients, respectively. However, lower cell-mediated immunogenicity to the same vaccine was recently reported in BTKi-treated CLL patients compared to patients with monoclonal B-cell lymphocytosis (MBL) or untreated CLL (37% *vs* 73%) (86).

In another study assessing antibody response to the 13-valent pneumococcal conjugate vaccine (PCV13) in 112 patients with CLL, of which 35 during ibrutinib treatment, immune responses were observed in only nine (8%) patients, eight treatment-naïve and one on front-line ibrutinib. None of the patients vaccinated ≥6 months after chemoimmunotherapy treatment with anti-CD20 monoclonal antibodies developed an immune response (87). Similarly, no adequate immune response to PCV13 was observed in 4 previously treated patients receiving ibrutinib while it was seen in all 4 previously treated CLL control patients with no ongoing therapy (88).

## Conclusion and Perspectives

During the last decade, treatment with BTKis has significantly improved the prognosis of R/R CLL patients. More extended clinical applications are also awaited, e.g. in the setting of first-line treatment in combination with other biological drugs, such as Bcl-2 antagonists. However, it is now clear that treatment with BTKis broadly affects the adaptive and the innate immune system in patients with CLL. This could explain the relatively high incidence of certain types of infections, such as invasive fungal infections, observed in ibrutinib-treated patients (48, 49). Infections are higher in the first months of treatment and the incidence decreases with decreasing tumor burden. However, when studying infection susceptibility in CLL patients, many factors should be taken into consideration which make it difficult to compare data from different studies. Among these, biological features of the disease (low *vs* high-risk) as well as previous and concomitant treatment including steroids, exposure to environmental risk factors, hypogammaglobulinemia with or without ongoing Ig substitution.

With regard to vaccination efficacy, the available data indicate that treatment with BTKi hampers the ability to mount a vaccine-induced immune response against novel antigens. Since T cells are also affected, at least by treatment with ibrutinib, it can be speculated that T-cell dependent B-cell activation is impaired twice, by BTK and ITK inhibition. This issue is of enormous relevance in the light of the ongoing COVID-19 pandemic, especially since patients with CLL may be at high risk of poor outcome if hospitalized due COVID-19 (89, 90). Further investigation is warranted, including studies on novel vaccination strategies, in patients with CLL. Indeed, it is presumable that patients in different disease phases and with different treatment histories (previous/ongoing) will not respond unanimously to vaccination, and that a patient-tailored vaccination approach might be needed, including temporary suspension of CLL treatment in patients in clinical remission to maximize vaccination efficacy.

Many new inhibitors, which bind reversibly, non-covalently, to the cysteine 481 of the BTK are under development. Indeed, due to safety concerns, irreversible inhibitors are regarded with caution as possible treatment for non-malignant diseases, such as autoimmune ones. Therefore, an alternative binding manner which would allow the compound to inactivate the BTK only temporarily, but with greater potency, is appealing. The expectations from these new compounds are also that they will not cause treatment-resistance due to BTK and/or PLCγ2 mutations. However, “off-target” effects are expected to be more consistent, due to the potential binding to multiple structurally related kinases. The challenge is therefore to design reversible covalent binding compounds with more tuneable, on-target residence times (91) than those observed with irreversible inhibitors.

In conclusion, further studies are needed to characterize in depth the biological effects of BTKis to implement new applications of the existing inhibitors in other disease settings or in combination with other drugs, and to guide the development of new, more or less targeted, inhibitors. Moreover, a deeper knowledge of the impact of BTKis on the immune system could guide clinicians in the management of infections and in the prevention thereof.

## Author Contributions

MP and TM wrote the first version of the manuscript, AÖ edited the first version of the manuscript. All authors contributed to the article and approved the submitted version.

## Funding

This work was supported by grants from The Swedish Cancer Society, Ref nr: 20 0726 PjF; The Cancer Society in Stockholm, Ref nr: 184203; The Cancer and Allergy Foundation, Ref nr: 258; Dr. Åke Olsson´s Foundation for Haematology research, Dnr: 2018-0015.

## Conflict of Interest

MP: Takeda, research support. Beigene, research support. AÖ: Beigene, research support. Astra Zeneca: research support. Gilead: research support. Loxo Oncology: research support.

The remaining authors declare that the research was conducted in the absence of any commercial or financial relationships that could be construed as a potential conflict of interest.
